# New Jersey State Dental Society

**Published:** 1889-09

**Authors:** 


### NEW JERSEY STATE DENTAL SOCIETY.

The nineteenth annual session of the New Jersey State Dental Society was held at Asbury Park, July 17, 18, and 19, 1889.

*Thursday, July 18.—Evening Session.*

Illustrated lecture by Dr. W. Xavier Sudduth, Philadelphia, on "The Individuality of the Biological Cell."

*Dr. Sudduth.*—The subject announced for me this evening is the "Similarity in Development of Hair and Teeth," yet I ask the privilege of referring to it only incidentally, and dwelling upon the individuality of the cellular structures that constitute all multicellular organisms, whether vegetable or animal. I base the assertion that the metazoa (many-celled structures) are an aggregation of protozoa (or single-celled organisms) upon three distinct phenomena, which I think I can clearly demonstrate to you this evening.

The first is the direct evidence to be drawn from cellular morphology.

The second is the similarity of the cells that constitute the formative layer of the rete Malpighii, to which latter I shall in the main confine my remarks this evening, and yet the dissimilarity of the resulting products that are developed from this layer, such as hair, sebaceous and sudoriferous glands, nails, horns, hoofs, scales, and the enamel covering of teeth, thus proving my third proposition, which is that each and every individual structure (cell) that forms a part of the formative layer has stored up in it an hereditary tendency which will eventually, when under the proper conditions, result in the tissue, the prototype of which must be contained in the formative element or cell.

But before proceeding with the lecture, it may be well to tell you how the photo-micrographs, with which the lecture will be illustrated, are made. In the first place, we prepare the tissues by cutting them in slices with a microtome, an instrument specially constructed for cutting very thin sections. The sections are then stained and mounted on glass slides, then with the aid of a microscope and camera they are very much magnified, and their image thrown on a negative plate. In this process the only work that is artificial, as you may say, is the hardening and the staining process. The idea is to get them in a condition in which they may be preserved and studied with as little change as possible. It is, however, impossible to prepare and cut them without some change or shrinkage. But we have brought our methods of hardening tissues to such a state of perfection latterly that there is very little noticeable difference between the tissues in their original condition and after they have been hardened. I will show you some pictures in which the hardening process has not been used; and there we virtually have the shadow of the living tissue. So far as accuracy is concerned, there is no method for demonstration to compare with that of the photo-micrograph. By this means we put upon the screen a representation of the original tissue without the intervention of man's hands, and as nearly perfect as it is possible to get it. You can see the shadow of the tissue and make your own diagnosis, and draw your own conclusions from what is on the screen. If I demonstrate to you with the microscope I cannot look at the specimen at the same time you are looking, and cannot see exactly as you see. The focal distance of different persons' eyes varies, and it is almost impossible for two persons to look at the same object and see in it the same aspect. But with photo-micrographs thrown on the screen you have the most accurate representation of tissue that can be made, in my judgment.

It is almost impossible to convey to the minds of those who are not conversant with histological work any idea of the innumerable numbers of infinitely small elements that constitute the body. I think I shall be able to show you that animal tissue is made up of innumerable units, which units bear the same relation to the animal system as the bricks in a house do to the whole structure; and that each individual element is a separate entity, and all are united by a cement-substance. We shall see that the human organism in all its complexity is simply an aggregation of individual organisms, each having a separate function to perform and an individuality to follow out. I will use the terms unicellular organisms and multicellular organisms, or the protozoa and the metazoa. The protozoa are unicellular organisms,—that is, bodies made up of individual units,—single bricks; the metazoa are multicellular organisms, —that is, bodies made up of many units, or many bricks united so as to form a wall.

It has been said that this line of study is not up to the latest date, that these terms are old and obsolete; but these terms are in general use, and we understand what they mean. We want to use common terms as much as possible, and not introduce new-fangled ideas and words that are not understood; we want a nomenclature of common language that we can use intelligibly. The text-books, including the latest one by Binnet, use the terms unicellular and multicellular organisms; therefore these terms are not obsolete in our text-books; they are used in English, French, German, and Italian books, and have the same interpretation in all these languages.

We have a class of organisms that are very low in the scale of life, at the very foot of the ladder, having no power of motion even, and which it is impossible to determine positively whether they belong to the animal or the vegetable kingdom. These are the micro-organisms that have given us such a wide field for study within the last ten years, and which study has done so much to explain the questions and solve the problems that have so long puzzled scientific men.

These unicellular bodies possess many of the qualities exhibited by higher forms of life. Binnet, a Frenchman, has recently written a book upon the psychic life of micro-organisms, in which he says that all or nearly all the phenomena exhibited by the metazoa may be distinguished in the protozoa. It is a most interesting little book, and I would advise all who have any interest in these occult subjects to obtain it. My own observation, to a certain extent, bears out many of the statements made by M. Binnet. Micro-organisms are living structures, possessed of the property of assimilation and multiplication, many cells arising by division of the original mass (fission), or by the throwing off of spores (sporulation), which ultimately reproduce forms of life like unto the parent cell, and functional activity, as seen in the production of ptomaines. In some of the higher forms sexual propensities are even described, and certain actions are interpreted as being veritable copulations. My own studies have not, however, been sufficiently extensive in this line for me to approve or gainsay the truth of the assertion. Reasoning from the known manifestation of sexual powers in animals,—only a little higher in the scale of being than micro-organisms,—we are fully prepared however, to accept the statement.

[Dr. Sudduth here showed on the screen a number of pictures representing micro-organisms,—the bacillus of tuberculosis, anthrax, swine-plague; blood-corpuscles and the tissues of the lower animals, and the human fœtus in various stages of embryonic life, all of which he explained as they were shown. A part of Dr. Sudduth's explanation is as follows]:

This is a photograph of blood as it was drawn on the slide and photographed, without staining or hardening at all. These individual elements or red blood-cells are entirely separate. They float freely in the liquor sanguinis of the blood. In form they are double concave disks. They are not united together. The round object in the centre of the field represents a white blood-corpuscle surrounded by red blood-corpuscles on the sides. The peculiarity of the white blood-corpuscle is that it has the power of locomotion; it can travel from one portion of the field to another. It has been called the human amœba. It consists of a mass of protoplasm which has the power of locomotion, and the power of digesting the elements of the tissues. The red blood-corpuscles have not the power of locomotion in the same degree that the white have, but they possess it slightly. I have watched these white blood-corpuscles for hours at a time. They throw out processes like legs (pseudopædia), little projections of protoplasm extending out from one side, and by means of these the corpuscle pulls itself along. These cells pass out through the interstices of the cellular wall,—through the mortar between the bricks. I want to call attention to the fact that we have individual elements which float freely in the liquor sanguinis, but which, when they pass out from the capillary vessels, become in a manner tissue-builders. It is true that tissue is developed, in a majority of cases, where there are few such cells, but the white blood-corpuscles are always more or less a factor in the development of tissue. It is easy to see that these are individual elements, that they stand in the same relative position to the structure as the bricks in the pile do to the completed building. As we put bricks in a house they become part of the house. The body is made up of many thousands of pieces, as is the house. Then we would say that we have individual units, and that these individual units possess in themselves certain characteristics; that they have the power of motion in some instances; that they have the power of performing function, of digesting tissue, and feeding themselves.

Here we have a section of the mucous membrane of the mouth. Here we have the individual elements, the units in the body. These tissues may be represented by the walls of a house. Each one of these cells that you see here represents an individual element, or brick. The dark lines are the counterpart of the mortar that holds each brick to its fellow. The nucleus, which shows here as a dark spot, is the vital portion of the cell; in these nuclei lies the formative action of the tissue, which is simply an aggregation of individual structures. The metazoa, or multicellular organisms, are made up of the protozoa, or unicellular organisms.

Here is a photograph of cartilage. Here we have the wall again, with its individual bricks. Each one of these individual elements represents the stones or bricks in the walls of the house. This cartilage is semi-elastic tissue, such as that which you find in fishbones and in the body of the ear. I next also show you hyaline cartilage, which consists of innumerable numbers of individual elements, lying in an undifferentiated basement substance. As you pass from this central portion towards the line of developing bone, you see the transformation of these tissues. Notice how, from being indiscriminately distributed, they gradually assume a perpendicular arrangement into columns, lines, or rows. There is order in all things. Tissues follow out certain well-formulated hereditary tendencies.

The next slide shows Meckel's cartilage. You see the individual elements as they go to make up this large mass. The forming bone is represented on the side, consisting of layers or lamellæ, upon the surface of which are seen the osteoblasts and in their walls the bone-cells. The osteoblasts are the bone-builders which build this osseous structure. Here you see these cells in the process of being built into the wall. As the bone is developed the cells occupy the spaces in the bone, as bone-cells, or the living part of the bone.

Here you have a cross section of a blood-vessel, representing an Haversian canal, and surrounding it are the osteoblasts.

This is the same, magnified very much higher. The osteoblasts are nuclear structures almost entirely, and are not typical cells, as has been generally conceded,—but I will speak of this more at length at some future time.

The next slide taken from the anterior portion of the spinal-cord, gives us another tissue, and which belongs to the nervous system. Here we have a composite organism, one that is made up of two kinds of tissue,—epithelial tissue and connective tissue, or cement-substance, composed of fibrous tissue. The epithelial cells have processes running off from them. This slide was stained with Bismarck brown, which is one of the best stains we can use for photographing.

Not all these cells have processes alike, for here we have cells without any and some with one or two processes, and some that have many processes. Here is shown the central nucleus, which differs decidedly from the basis substance.

Our next slide is an excellent preparation, made by Dr. Andrews, and consists of a highly-magnified photo-micrograph of developing dentine, showing the odontoblastic layer upon the surface where the cells have been mechanically separated. We find that when we tear the tissue apart the odontoblasts hold to the dentine more or less firmly by their processes, which extend into the dentine on one side. You can see quite plainly that they are individual elements.

Our next slide consists of a section of the skin. At the deepest portion we see the cells developing. The vascular supply is found beneath in the true skin; no blood-vessels having ever been demonstrated in the epithelium.

Here we have the infant layer shown upon the screen, very highly magnified. A cell has a nodal point, with the protoplasmic material surrounding it. It is proven that the vital portion of the cell is found in this central point. In embryonic structures these lie in what are called naked cells. These central nodal points lie in an undifferentiated bed of protoplasm. Protoplasm has the power of transmitting sensation and of performing function. In certain animals it serves the purpose of a nervous system; and this property of protoplasm accounts for the sensation in the dentine. This is brought out very plainly in a late work of Binnet's on micro-organisms. I hold that it is not necessary that we have differentiated nerve-fibre in order that sensation may be transmitted from one point to another. I have, however, been criticised on that point.

The development of these animal cells is by fission. It has been ascertained that these granular particles (nucleoli) become polarized, and arrange themselves in opposite ends of the cells; and when these granular particles collect in either end of the cell then we have a constriction of the cell; then the cell divides into two, and it is divided again and again in geometrical progression. There are other ways by which tissues grow, and one is by sporulation, as is shown in the next slide, the bacillus of swine-plague (hog-cholera). We have here the central portion of the cell divided into two spores. The protoplasm is thin, and transparent between these two points. They are like two beads joined together. I have seen, and caused by special methods of culture, anthrax bacilli to develop into long chains, and then form spores. This is another triumph of the investigator. He can at will produce this division of tissues. If you give the anthrax bacillus its natural food it grows very rapidly indeed. We are able to see the different changes in the tissues. Then again, we have the development of lower forms of life by a similar process of those of the tissues of the body. The amœba, by splitting off a portion of itself, develops another amœba. In the development of the tissues that go to make up the body, the original impulse of the development must come from the nucleus itself.

Now, studies have been made upon the Stentor cœrrulous, an infusory which represents a very low form of life, but which is of sufficient size, so that by placing it under a microscope and then using extreme care you are able to divide it into different parts. If we simply cut off a portion of the protoplasm, then we find that the severed portion will live for six or eight or ten hours only; but if we divide it in such a way as that some of the nuclear portion goes with the protoplasm, it will produce another infusorium with all its essential parts. It has the power of motion after division for a limited period of time, but has not the power of producing one like itself without some of this nuclear portion. The real life-giving force does not reside in protoplasm as a mass, but in the central nuclear portion. Protoplasm has the power of digesting tissue, but it has not the power of instigating propagation or reproduction; that power lies in the nodal point or nucleus, in the central portion of the cell.

It has also been found, in these protoplasmic masses, which have no outside limiting wall or cell-wall, that the protoplasm itself will throw out little feelers, feeling for a place to attach themselves and pull the body over; or in searching for food, as some of the higher amœba do search for food. We have that action which is a living action in this material, but this one material only acts by instigation of the central point. The nodal point or nucleus is the point from which the impulse springs.

We have shown pretty clearly the character of isolated cells; now we desire to call your attention to cells *in situ*, and by special methods of technic demonstrate that many cells of the body do not form a solid mass. This is shown by the next slide, which is taken from the mesentery and is stained with nitrate of silver. These dark lines mark a division between each and every cell. Each one of these points represents an individual cell, with its nucleus in the central portion. They form a single layer of cells, and when so united are called endothelium; they form the covering of all closed cavities and the lining membrane of blood-vessels. It is between these cells, in these dark lines, that we have an egress for the white blood-corpuscle.

We have very fully considered the morphology of cells; let us now turn our attention to the evidence which may be drawn from the different phases of development and see if we can throw any light upon their character from this point of study.

For our next slide we have the ovum or egg in its initial development. It consists of a single cell, made up of a granular layer surrounding the vitellus, which corresponds to the cell-body and containing a nodal point which compares with the nucleus of tissue-cells. When the ovum is ripe the tissue over its surface breaks open and allows it to escape. All ova have more or less the same morphological appearance. From this initial cell we have the development of all the tissues. We will trace the hereditary tendency that is pent up in these individual elements.

The next slide shows us a drawing of a human ovum. Heretofore all our studies in the development of ova have been confined to those of animals lower than man. Within the last few years we have been able to make studies of human ova; and what you see is a drawing made by Nagel, representing his observations upon the human ova at a clinic in Berlin. Taking the warm ovum, after it had been obtained in the operation of laparotomy, he found that the division and the changes which occur in the human ova are almost identical with those that occur in many of the lower animals; and this fact has brought us to a point decidedly in advance of our former knowledge regarding division of tissues in the human system.

The next slide represents the ripe ovum which is cast forth. He found that in the central portion polar globules were formed similarly to those in rabbits and cats. Division begins in the nodal spot; and the subsequent changes of the body surrounding are the result of the governing force that comes from this portion.

The next slide is also a drawing. It shows how these cells divide. Here you see division of the nucleus into two parts. The process goes on until there is a separation of the entire ovum. Here are spermatozoa. As fecundation progresses we have a new structure, the result of the union of these two elements. If you say that the ovum is passive, and that the reproduction probably comes from the spermatozoa, I will take no exception to that. But we have in this ovum and spermatozoa the potentiality of the future product.

In the ovum and spermatozoa must lie the hereditary tendencies which will by future growth give us the several organs and tissues of the body, for they do not form until later on, but in their development precede function, so that they cannot be said to arise as a result of function. I think that I can demonstrate to you that by division of the original cell we have a division of function. The original cell divides, as here shown, first into two, then into four, then into eight, sixteen, and thirty-two, and so on, until we have a sufficient number of individual elements, or kinds of cells, to represent the different functions of the entire system.

The next slide shows us how by division and differentiation we have the development of the three layers,—epiblast, mesoblast, and the hypoblast. From these three layers the different tissues of the body are developed.

We never have any interchange between the epithelial elements and the mesoblastic elements. They remain throughout the life of the individual entirely different. Why it is that by differentiation from one original cell we have these different layers formed, which have entirely different functions, I cannot tell you, but such is the case.

The next is a photograph of the epiblast as seen under the microscope with a high power. We have here the individual elements,—the bricks in the wall. The life of the individual lies in these nodal points, and from these points go out the active functionizing powers of the tissues. They are shown here lying in an undifferentiated basement substance. As we follow these out in later stages we see that this part, the inner layer, becomes thickened; these nodal points develop around themselves a certain amount of formed material.

If we take any of these cells and transplant them on a living tissue they will grow. Dr. Garretson for several years experimented in that direction with cancer. He first took scrapings from the arms of students, until they grew tired of it. He finally resorted to curryings from horses, and transplanted the epithelial cells so obtained to raw surfaces, and they grew. I saw in clinics at Vienna, especially in the skin clinics, the transplantation of small pieces, which made a new growth and adapted themselves to the new tissue where they were placed, showing that scrapings of the epithelial tissue from the surface may be revivified if they contain this central or nodal point. Unless the portion cut off contains nuclei, it will not grow, but, as has been shown by experiments on lower forms of life, it may live eight or ten hours.

We know that from the skin are developed hair, sweat-glands, fat-glands, the nails, horns and hoofs, so-called dermal appendages. These structures are developed by proliferation of the cells of the infant layer of the epiblast.

Here, on the screen, we have the first stage in the development of a hair. Here is the Malpighian layer. At this point there was a rapid development of cells which caused a thickening of the deepest layer of the epiblast; and by that thickening there resulted a depression of the layer into the tissue beneath and the formation of a bud. As this goes on you will find that the next stage shows that process dipping down farther into the tissue. The most rapid development of cells is at the deepest point, while the overlying cells remain apparently the same, except that the skin becomes a little thicker as age advances. Thus it appears that the glands, the hair, and the enamel organ of the teeth are developed by proliferation of one or two cells that form solid growths into the tissue. The differentiation lies in the cell itself. We have no power to see whether a particular cell will develop a hair, a sweat-gland, a fat-gland, or the enamel organ of a tooth. In the nodal point lies the history or record of what it is going to be.

Here we have that bud dipping down farther into the tissue, and a condensation all around the side, and forming the connective tissue-envelope. The bud dips into the tissue a certain distance, and then these secondary changes occur. The hairs of the face are much deeper rooted than the hairs of the head. The feelers of the cat dip away into the tissue, while the other hairs of the cat only go slightly under the surface. We have no means of knowing why this is. That individuality also lies in the nucleus. By this division and elaboration we have a differentiation and division of function as the result of hereditary tendency.

The next step in development is the formation of the papilla. This papilla carries with it a vascular supply that will make the root of the hair.

Here we have condensation of the papilla in the formation of the root of the hair. Here you see the sebaceous gland forming. Here we have a hair developed, but much less highly magnified. This is taken from a child three days old. The first hairs developed are called lunago hairs, and are only temporary, being shed and replaced by more permanent hairs. A bud is thrown off from the base of the first hair, which is crowded out, and a new hair takes its place. That is exactly analogous with the development of the permanent tooth from a bud that comes off from the side of the temporary tooth. The development of a hair seems to be quite a simple process. We are not prepared to say that there is no design in regard to the placing of hairs; although they dip into the tissue apparently without any particular order of arrangement. In certain animals, however, as in the cat, certain hairs are used as feelers. There is apparently a place or point where these hairs are to go down; but it is not so plainly marked as with the teeth. In the shark the spines of the skin are differentiated into its teeth. The mucous membrane of the mouth is identical with the skin on the outside. In the development of human teeth, and the teeth of all the carnivora, we find design as to location. Take a cross section of the jaw of a pig; here you have the grooves, so-called, dipping into either side of the jaw; on the other side of the cross section there is the epithelial layer which constitutes the skin. In the embryo the cells in both are morphologically identical.

This is a longitudinal section, showing the outline where the teeth are to be developed. This is called the band which may be said to be common to all the temporary teeth. Hairs, however, come directly from the surface without the interposition of any point or band which in the case of the teeth determines their location. But for that we should have teeth growing all over the jaws, inside and outside and everywhere. This is broken up into the outer and the inner lamella. From the inner lamella we have put off the cord, that corresponds with the bud in the development of the hair. There is an epithelial bud dipping down into the tissue. The differentiation is always at the deepest point, commencing in a single cell which, by multiplication, causes at first a slight thickening of the rete Malpighii. As the bud goes down you see a constriction of the neck, and the cord becomes bulbous.

Here we have a cross section of another jaw, showing the development of the jaw; and here the developing teeth on either side, and the first development of bone. Here is the first indication of the development of the connective-tissue periosteum surrounding the jaw. Here you see the breaking away of the mother layer, and there the papilla is beginning to take a conical form. There is a portion of the stellate reticulum of the enamel organ. Here the alveolus is seen developing on the side; there the papilla is assuming the form of the future tooth; here the cord for the development of the permanent tooth, just as the cord was in the development of the lunago hair, or for the formation of the permanent hair.

I have tried, although imperfectly, I know, in the time allotted, to show how from a common starting-point in one cell, by division, the potentiality stored up in the mother cell was divided into the several cells, which afterwards served as the nodal points from which went out the impulse that resulted in the development of the several organs and parts of the body. Organization in the embryo certainly precedes function, for we find the several parts completed before function begins. We cannot, however, doubt that environment largely controls the formation and selection of organs, and we can only explain the phenomena found in developing tissues by accrediting to each element a separate entity, and admitting it to be possessed of an hereditary tendency which will result in the production of tissues in accordance with the typal demands of the parent from which the original cells, ovum, and spermatozoa sprung. No one will be bold enough to hold that the father has any direct influence upon the developing fœtus except through the mother; and few claim that the mother has much influence over it during fœtal life, and certainly none in the oviparous fish and fowls; for instance, where the ova, after being impregnated, are cast off to look out for themselves, and are not brought under the vital control of the parent; and yet the type is always reproduced even more faithfully than in viviparous animals. Now, we must admit, from the phenomenon of development as well as the morphological appearances of tissues, that the body is composed of innumerable elements, termed cells, which have stored up within themselves distinct hereditary tendencies, which in some cases develop a hair, another a gland, and still another the enamel organ of a tooth, according to the typal demand found in the cell itself.

The slides are at the disposal of any one desiring to speak on the subject, and I hope they will ask for any specimen they wish to speak upon.

On motion of Dr. Watkins, a vote of thanks was tendered to Mr. Smith for his kindness in giving the magic latern exhibition.

*Dr. Carl Heitzmann.*—Mr. President, we have been treated to a large number of drawings, or photo-micrographs, and a still larger number of words. All of which, I suppose, was extremely interesting to every one who knows a little of the microscope and does not penetrate very deeply into the subject of the formation and character of the tissues. It is a strange thing, indeed, to see how few people are able to do some thinking of their own. The brains of most people are like street organs, which are capable of playing certain tunes only; whenever the machine is wound up and let loose, nothing but these tunes are played over and over again. So it is with some persons who have been impressed by reading books and listening to the teacher's words. It is fifty years since Schwann started the cellular doctrine; and, of course, it clings to the brains of the majority of people in our day, even after all the efforts of some sceptics, who have tried it faithfully and found it of very little value. The doctor speaks of cells, referring to the text-books. Why should the cell-doctrine be correct? Because the books say so? May I ask the essayist whether he is able to give a definition of a cell? I am sure he will say no. Nobody knows; and certainly I do not expect it from him, because bright men have made futile efforts to give a definition of a cell. The best definition is given by Drysdale, of London, who says a cell is a gun without lock and barrel. And this indefinite thing that we call a cell is the basis still of modern histology.

We know that the smallest piece of living matter is able to grow and to move; and thus will do everything that a large piece can do, such as an elephant. I am sure the word "cell" will not add the least particle to our knowledge of living matter. Such a thing as the cell does not exist in the tissues. It has been proven by myself, seventeen years ago, that within the tissues of the human organism there are no individual cells. I am surprised that

Dr. Sudduth thinks that work is not even worthy of mention. I have, by real hard study, shown that what the profession has been told was made up of individual cells is uninterruptedly connected throughout the tissues. Why does the essayist ignore it? My views have been endorsed by a number of good men, such as Abbott, Bödecker, Atkinson, and a score of others, who are convinced that there are no individual cells whatever in the tissues of the body; that what are called individual cells are, in fact, uninterruptedly connected, and that the intervening mortar, as the essayist calls it, is just as much alive as the so-called cells themselves, for all that we can find out. Why should Dr. Sudduth ignore what Stricker has said since 1880, and which is entirely opposed to the cellular theory, that there are no individual cells in the tissues, and that the protoplasm has just as much life as the basis-substance?

Since about eight years the botanists have come to our rescue, and now support the same theory. There is not a single botanist of note who is not convinced of the fact that what has been called the individual cell is not at all individual, but that throughout the cement-substance, or cellulose, there prevails a continuity by threads of living matter, piercing the apparently inert cement-substance, and that all plants are but one continued individual from the ends of the rootlets up to the points of the leaves,—and that is just what I have claimed seventeen years ago in relation to animal organisms. The tissues, therefore, are not made up, as Dr. Sudduth says in rather queer English, of innumerable numbers of cells which may be considered individuals, but are continuous masses of protoplasm. It is sad that seventeen years' effort on my part has left me almost alone in this assembly, with the exception, perhaps, of Dr. Atkinson. We know very well that such efforts, although just the opposite to the current street-organ tunes, are hard to comprehend and hard to accept. If I had happened to be born a century earlier I would have been burned at the stake or stoned to death for my heretic teachings. To-day I take it easy, because my conscience is clear. If I maintain anything, I can show it,—not with photomicrographs though. That is a poor way. The photograph cannot give a correct idea of the structure of the tissues; you must study them with the microscope directly, and then form your own judgment from what you see. Histologists have discovered very wonderful things, and Dr. Sudduth especially discovered the naked cell. I suppose his next discovery will be cells with pants on.

As to the similarity in formation between the hair and the enamel organ, or rather the enamel cord, that was shown forty years ago by Todd and Bowman, who, in their celebrated cyclopædia, have asserted that teeth are tegumentary formations, on account of the similarity of origin of the hair and teeth. But just as sure it is that the similarity between the epithelial formation of the hair on the one side, and of the enamel organ on the other side, is very slight. The enamel cord begins to show in the sixth week of embryonal life; the hair in the twelfth. The hair is epithelial in its origin, and remains epithelial throughout life; the enamel organ, on the contrary, is epithelial only so long as it is a cord; as soon as the cord broadens to the enamel organ it ceases to be epithelial tissue. In the first edition of A. Kolliker's work on histology, issued in 1852, he claimed that the enamel organ is connective tissue; yet much later—I do not know from what cause—he changed his opinion and held that the enamel organ is epithelial throughout. So long as he was unprejudiced by the later writers,—Remak, His, Waldeyer, etc.,—he took the enamel organ for what it is, a reticulum of connective tissue. At first the similarity of growth between the hair and the enamel organ is striking, but as soon as the enamel cord develops into the enamel organ it ceases to be epithelium; it changes to medullary tissue, and finally forms the enamel.

In conclusion, I wish to draw attention to two mistakes that Dr. Sudduth made; one historical, the other grammatical. The historical mistake is that he said Pasteur first described the anthrax bacillus. No; that was Davaine, in 1863. The grammatical mistake is where he spoke of the Stentor as being infusoria. In the Latin Stentor is singular; therefore infusorium.

*Dr. Atkinson.*—I do not think, Mr. President, it is at all worth while for me to make any remarks before this body. If there were five or six of us to sit down, with a nice bottle of champagne, and talk this matter over, we might arrive at something. This question has been discussed in various societies by gentlemen who think they discussed it, but who did not comprehend the first postulates of what the difference between them is. The difference is more a matter of verbiage than of doctrine. That is the reason that it is so difficult to define what a cell is. I am astonished that men so strongly marked with the love of truth will not accept what Professor Heitzmann has brought forward, but still remain in a persistent effort to regard their nominations as of more value than the advanced truth that is couched in the better language of Professor Heitzmann. They persist in using the name cell, utterly refusing to define the thing intelligently. Every single pathologist has committed that error, from Schwann down to this day. They have given us simple cells, nucleated cells, nucleolated cells, and now we have naked cells. That is a necessary result of a bad beginning. I am so much of a baby as to like to hear the little lispings and see the efforts of the growing mind to tell what it has found. I feel that a baby is the divinest thing on the planet, and this is our baby.

I won't talk about the grammatical part of it. I concede that it is better for a mixed and general audience to demonstrate with one specimen by photo-micrographs, so that all can see the identity of that which is presented. Here we have it represented on the screen the same to all, only in the difference of stand-point that we occupy; by which method there is less ambiguity than there would be were each individual to look through the microscope for himself, by reason of the necessity for the adjustment of the focus to different eyes.

How is it that we get the germ at all? There has been nothing said about that. But if we have to go farther, I have no doubt Professor Sudduth would give us in a very lucid manner the metamorphoses through which the two semens go; the ovum standing as one, and the spermatozoa standing as the other postulate; then they must have their potentialities awakened so that they combine into a homogeneous mass, unlike either of them, and which is now a prepared germ, holding within it the class, genus, species, and variety of the parent stock from which it was taken. It appears under the microscope as nothing but a cloudy mass, which has lost its former characteristics, and is now differentiated into the epiblast, hypoblast, and mesoblast; and then they enter into a quarrel as to whether the mesoblast has a different origin, or whether, in the proliferation of these indefinite corpuscles,—you see I use the term corpuscle; they are bodies,—some of these shall come from the epiblast, and others from the hypoblast, and then become the basis-substance of the splanchnic walls and body-walls of the organism that is of the same typal demand, according to the class from whence the protozoa were derived; holding all these potentialities during the metamorphoses, and during the storing of the radiancy that constitutes the starting-point of each one of the granules, the accumulations of which constitute the indifferent bodies known as indifferent corpuscles, embryonal corpuscles, multinucleated and giant cells, of which the bone-plates, nerve-plates, and muscle-plates consist, from which the tissues arise by due increment and appropriation of the stored radiancy, according to the typal demands; first corpuscles, then tissues, then organs, then systems, according to the types of the stock from which they came.

If we would correlate our ideas into a clean understanding of what is atomic life, molecular life, corpuscular life, tissual life, systemic life, and conscious life, we would be able to get a syllabus that would be quite cognate with all the apprehensions of each individual discoverer, so that each should use his own term as a first nomination, and then give different terminations to indicate, in adjective form, as prefixes or affixes, whatever differentiations were made, so as to get rid of these difficulties, and then see eye to eye, and travel together with more profitable progress.

But after all, what is the difference? Each individual does understand something of what he tries to see; and the more earnest the man is the more liable is he to have a sort of paternal affection for his own nominations, and he may be even weak enough to run the thing into the ground by being determined to have everybody follow his nominations. I have no interest in the matter further than to get at the best way to apprehend and promulgate the truth.

Adjourned to half-past nine o'clock to-morrow morning.

### *Friday, July 19.—Morning Session.*

DISCUSSION OF DR. SUDDUTH'S LECTURE TAKEN UP.

*Dr. Sudduth.*—Mr. President, it would be hardly possible to add anything to what was said last night, without having the slides present to enforce what was brought out. I will simply say that I am willing to allow the case to go before the jury with the presentment I have made on the screen, as opposed to the discussion of the lecture on the other side. You have to carry away in your mind's eye the visible shadows of those tissues, and that will perhaps reinforce somewhat your memory in regard to what was said. The lecture I gave last night was a continuation of one on the same subject a year ago, when we took up general embryology; this year we have considered cellular morphology and the hereditary tendencies that reside in the tissues.

One or two things that were said last night were rather amusing to me. I am sorry that Dr. Heitzmann is not present to hear my reply.

In regard to the term "naked cell," to which he took exception, I will say that I was hunting around for words that would be simple and understandable, and so avoid technical terms; and I used a term that Zeigler had used to indicate a mass of protoplasm containing a nuclear structure without a cellular limit or wall; that he called a "naked cell." Zeigler is perhaps the most extensively read pathologist in Europe, and he used the term "naked cell" to express the infant cell. It was not new with me, but came to my mind as I was talking, and I used it in that connection. I do not want to force it upon you at all and do not propose to.

Another point was Dr. Heitzmann's correction of an alleged grammatical slip. That was really amusing to me. If Dr. Heitzmann were more conversant with English he never would have fallen into the error he did. There are quite a number of the Stentor family. Dr. Peirce will tell you that he has traced ten or fifteen different varieties of them. I said Stentor infusory, to signify, as it does, one of the Stentor family. Webster defines infusory as a noun, plural, infusories, one of the infusoria; an infusorial animal; and that is the sense in which I used the word. It was amusing to me that Dr. Heitzmann should have allowed himself to have been tripped up in that way.

What I said in reference to Pasteur was that he had done more, perhaps, in the study of anthrax bacilli than any other man; and that is a fact. While Pasteur may not have really first discovered the anthrax bacillus, he has done more towards its cultivation and study than any other man, and he deserves the honor. I was particular to say that any slide which the gentlemen wished to speak upon would be produced if they would call for it. He did not ask for a single slide, but went into a few generalities and personalities. When a man has to descend to such a line of argument as did Dr. Heitzmann last evening, he must have a very poor case indeed. Several remarks which he made in his speech were a sufficient answer to his own position. He said that Schwann proposed fifty years ago the name cell, and that it had continued in use until now, with but little change, and that he (Heitzmann) stood almost alone, with two or three of his friends and students, in opposition to Schwann. That seems to me a complete answer to his objections to the use of the word cell. It has stood through fifty years, and is used to-day almost exclusively in the text-books. That is sufficient proof that it is good enough to keep, and I do not propose to drop it because Dr. Carl Heitzmann does not like it. He spoke sadly of the fact that he had been seventeen years striving to get his theories accepted; and he had not got them any farther than the few he referred to. There was his own evidence that the presentation he nas given has not been accepted by the scientific world; and now he is trying to pose as a martyr before the world, saying he would have been burned at the stake if he had lived at an early day. The fact is that he has had the most kindly treatment by those who have opposed him, and he himself is the man who has been dogmatic and intolerant, and tried to browbeat those who opposed his theories. He is the twelfth juryman who abuses his eleven associates because they disagree with him.

As to Dr. Heitzmann's personal and wholly uncalled-for attack at the banquet last evening I have nothing to say, as I had not then. It is only in keeping with his idea of scientific discussion, and can only best be met with silent contempt.

The lecture was long and tiresome, and I fully appreciated the attention that was paid to it. I am pleased to be with this society; I always enjoy my trips down here; and if I can at any future time be of service to the society in this connection, I will be glad to serve you.

